# Comparative proteomic analysis of edible bird’s nest from different origins

**DOI:** 10.1038/s41598-023-41851-6

**Published:** 2023-09-22

**Authors:** Xianyang Wang, Dingwen Hu, Feng Liao, Sitai Chen, Yu Meng, Jie Dai, Tina Ting Xia Dong, Zizhao Lao, Liangwen Yu, Yueliang Liang, Xiaoping Lai, Karl Wah Keung Tsim, Geng Li

**Affiliations:** 1 State Key Laboratory of Traditional Chinese Medicine Syndrome, Guangzhou University of Chinese Medicine, Guangzhou, China; 2 College of Life Science, Wuhan University, Wuhan, China; 3 Division of Life Science and Center for Chinese Medicine, The Hong Kong University of Science and Technology, Hong Kong, China; 4 Mathematical Engineering Academy of Chinese Medicine, Guangzhou University of Chinese Medicine, Guangzhou, China; 5 Tunghong Medicine Co., Ltd, Guangzhou, China

**Keywords:** Phylogeny, Protein analysis, Proteome informatics

## Abstract

Edible bird’s nest (EBN) mainly made of saliva that secreted by a variety of swiftlets is a kind of precious traditional Chinese medicine. EBNs from different biological and geographical origins exhibit varieties in morphology, material composition, nutritive value and commercial value. Here, we collected four different EBN samples from Huaiji, China (Grass EBN), Nha Trang, *Vietnam (Imperial EBN)* and *East Kalimantan*, *Indonesia (White EBN* and *Feather EBN)* respectively, and applied label-free quantitative MS-based proteomics technique to identify its protein composition. First, phylogenetic analysis was performed based on cytb gene to identify its biological origin. Second, a total of 37 proteins of EBNs were identified, among which there were six common proteins that detected in all samples and exhibited relatively higher content. Gene ontology analysis revealed the possible function of EBN proteins, and principal component analysis and hierarchical clustering analysis based on 37 proteins were performed to compare the difference of various EBNs. In summary, our study deciphered the common and characteristic protein components of EBNs of different origins and described their possible functions by GO enrichment analysis, which helps to establish an objective and reliable quality evaluation system.

## Introduction

Edible bird nests (EBNs) are nests built from objects secreted by the sublingual glands of golden swifts^1^, which have high nutritional, medicinal and economic values and have become a very important industry. EBNs are distinguished by the color of their appearance and can be divided into external white nests, black nests, and red nests (or blood nests)^2^. Of these, red nests (or blood nests) are considered to have higher health benefits and are more expensive in the marketplace than other nests^2,3^. Modern pharmacological studies have found that edible bird's nests have pharmacological effects such as enhancing human immune function^4^, antioxidant^5^, moisturizing^6^, anti-wrinkle^7^, anti-skin inflammation^8^, improving memory in mice^9^, and anti-viral^10^, which further increases the market demand for EBN.

However, the high price and huge market demand for EBN have brought great challenges to the quality control of EBN. Counterfeit and adulteration are two sources of poor quality EBN. Pigskin, white fungus, starch, agar, etc. are frequently reported materials for counterfeiting fake EBN^11^. Mixing products with fakes or with EBN from different origin creates another kind of low-grade EBN as the quality of EBN is deep related to its biological origin and production location^12^. EBNs from different origin exhibits different commodity value^13^. For example, EBN produced in Hoi an, Vietnam sells for about HK$46/g, which is four times as expensive as grass bird's nest, which costs HK$10/g. Microscopic identification and morphological identification are often used to determine the authenticity of EBN^14^, but they are weak to distinguish similar EBN products that are cleaned and processed into fragments. Chemical composition analysis technologies, such as ultraviolet chromatography, infrared spectrometry, gas chromatography, protein electrophoresis and content determination of chemical constituents^13,15,16^, detect the difference of chemical composition to identify the authenticity and purity of EBN, but these methods are unable to provide reliable evidence for the identification of biological source and origin of EBN^17^. Therefore, a more reliable identification method is urgently needed.

EBN consists of 62.0% crude protein, 27.3% carbohydrate, 7.5% water, 2.1% inorganic ash, and 0.14% lipid^18^. Though protein accounts for more than half of the total materials, there are limited assays about the protein composition of EBN. Quantitative MS-based proteomics aims to measure protein abundance variations in protein abundance of proteoforms in different conditions^19^. Here, we collected four EBN samples from three major producing countries and applied DNA barcoding and Label-free quantitative proteomics approach to decode the different protein conditions of EBNs in a variety of origins, and our results show a potential capacity to distinguish EBN levels especially in processed and similar EBN and help to illuminate the function basis of EBN.

## Result

### Sample collection

**Figure 1:**
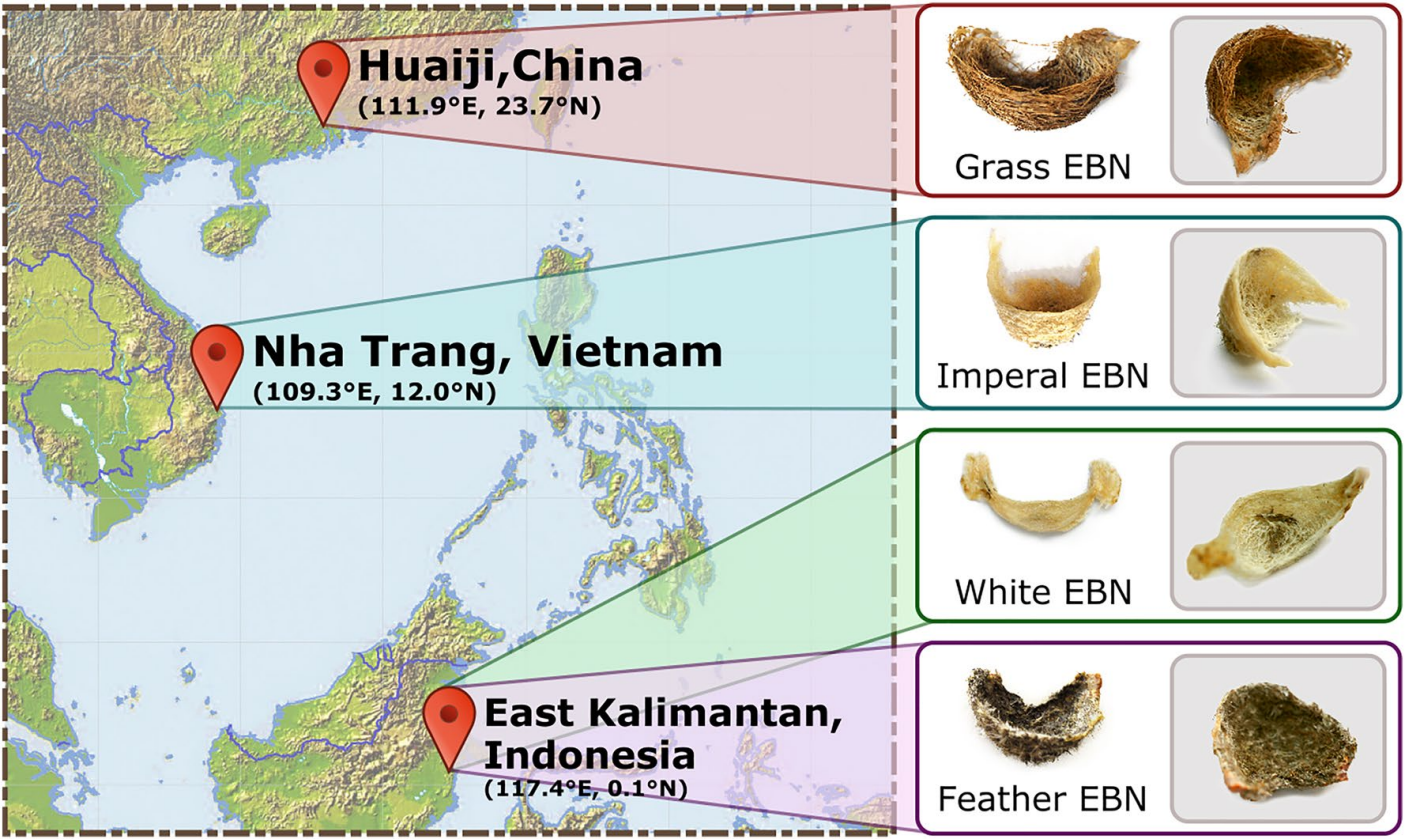
Geographic and morphological information of EBN samples. Grass EBN was collected in Huaiji, China. Imperial EBN was collected in Nha Trang, Vietnam. White EBN and Feather EBN were collected from East Kalimantan, Indonesia. The morphology of four EBN samples are shown in the picture.

Four EBNs were collected from three major producing countries, China, Vietnam, and Indonesia (Fig. 1). EBN samples collected from Huaiji, China are interwoven with grass fragments and show obvious saliva adhesion marks, which is identified as *Grass EBN*. The EBN sample collected from Nha Trang, Vietnam is identified as *Imperial EBN*, which is white and cup-shaped with obvious net-like interlaced saliva adhesion marked inside. The outer surface is smooth with layered parallel textures and there are only a few feathers on the bottom of the nest. There are two kinds of EBN collected from East Kalimantan, Indonesia, one identified as *White EBN*, and the other identified as *Feather EBN*. The appearance of *White EBN* is similar to that of *Imperial EBN*, but a litter longer and firmer. While the *Feather EBN* is bowl-shaped and dark gray, and there is a large number of gray hairs mixed and interwoven, and interlaced saliva adhesion marked inside.

### Genetic identification of EBN samples

**Figure 2:**
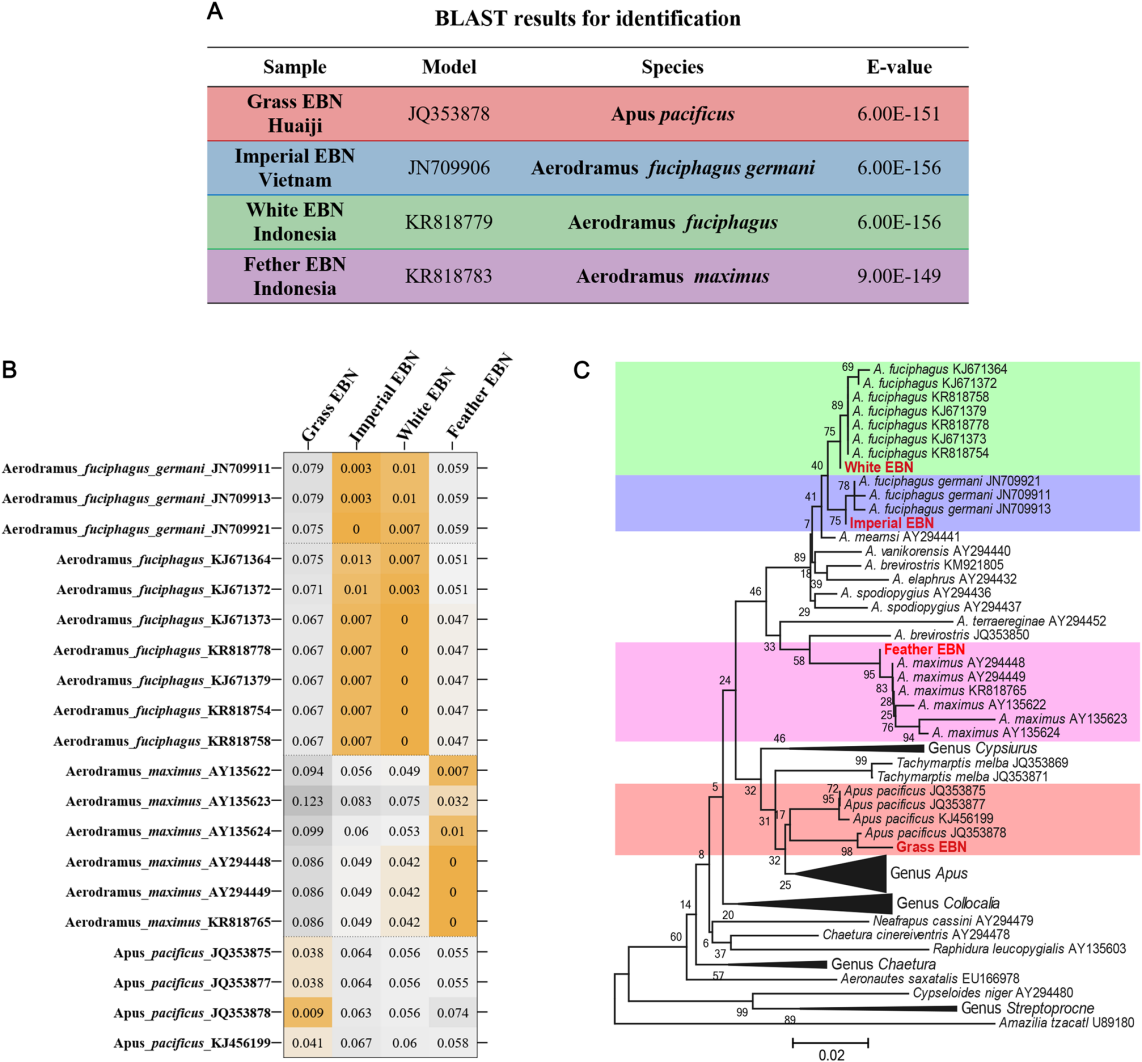
Genetic identification of EBN samples. (**A**) BLAST results of the four samples were tabulated and the login numbers of the sequences that most similar to the samples were marked as Model. (**B**) Genetic distance between samples’ sequences and model’s sequences. (**C**) Neighbor-joining tree based on cytb gene sequences of samples and *Apodidae. Amazilia tzacatl* was selected as outgroup. Sample sequences were marked in red font in the tree. The bottom blocks of different colors represented different species, and the bootstrap values were marked on the branches.

EBN is composed of solidified saliva secreted by the male swiftlet, while different swiftlet species construct diversified EBN in morphology and component^20,21^, leading to a wide difference in nutritive and commodity value. Before proteomic analysis, we first conducted genetic identification to confirm the biological origin of EBN samples using our previously developed method^22^. In a nutshell, complete genomic DNA was isolated from EBN samples, and mitochondrial cytb gene fragments were amplified using the nest PCR technique. DNA fragments were sequenced, and the outcomes were utilized to perform BLAST searches and calculate genetic distances. The BLAST result demonstrates that the sample sequences are highly similar to the cytb genes of *Apus Pacificus/Aerodramus* fuciphagus *Germani/Aerodramus* fuciphagus and *Aerodramus* Maximus, respectively, and that the sample sequences are homologous to the cytb gene (Fig. 2A). *Apus Pacificus/Aerodramus* fuciphagus *Germani/Aerodramus* fuciphagus and *Aerodramus Maximus*, respectively, are further evidence that the genetic origins of *Grass EBN, Imperial EBN, White EBN* and *Feather EBN* are *Apus Pacificus/Aerodramus* fuciphagus and *Aerodramus Maximus*. This is also supported by the fact that the Kimura 2-parameter pairwise distance between samples' (Fig. 2B).

We performed a phylogenetic analysis using a combined dataset, which included amplified cytb gene sequences and cytb gene sequences of *Apodidae* acquired from GenBank, to determine the relationship between species relatedness and the morphology and elements of EBN. Using the Kimura 2-parameter model, a neighbor-joining tree was created, with *Amazilia tzacatl* chosen as an outgroup. With bootstrap values collected 1000 times, the topology's dependability was assessed. The phylogenetic tree shows that the *White EBN* sequence is more related to the Imperial EBN sequence, which clustered with the *Aerodramus* fuciphagus and *Aerodramus* fuciphagus *Germani* sequences, respectively (Fig. 2C). While the sequence of *Grass EBN* is clustered with the sequences of *Apus pacificus*, the sequence of *Feather EBN* is with the sequences of *Aerodramus Maximus* (Fig. 2C). Collectively, from a genetic origin view, *Imperial EBN* and *White EBN* show similarity, *Feather EBN* is relatively similar to *Imperial EBN* and *White EBN*, and *Grass EBN* is highly diverse from others.

### LC–MS/MS analysis of EBN crude protein extract

**Figure 3:**
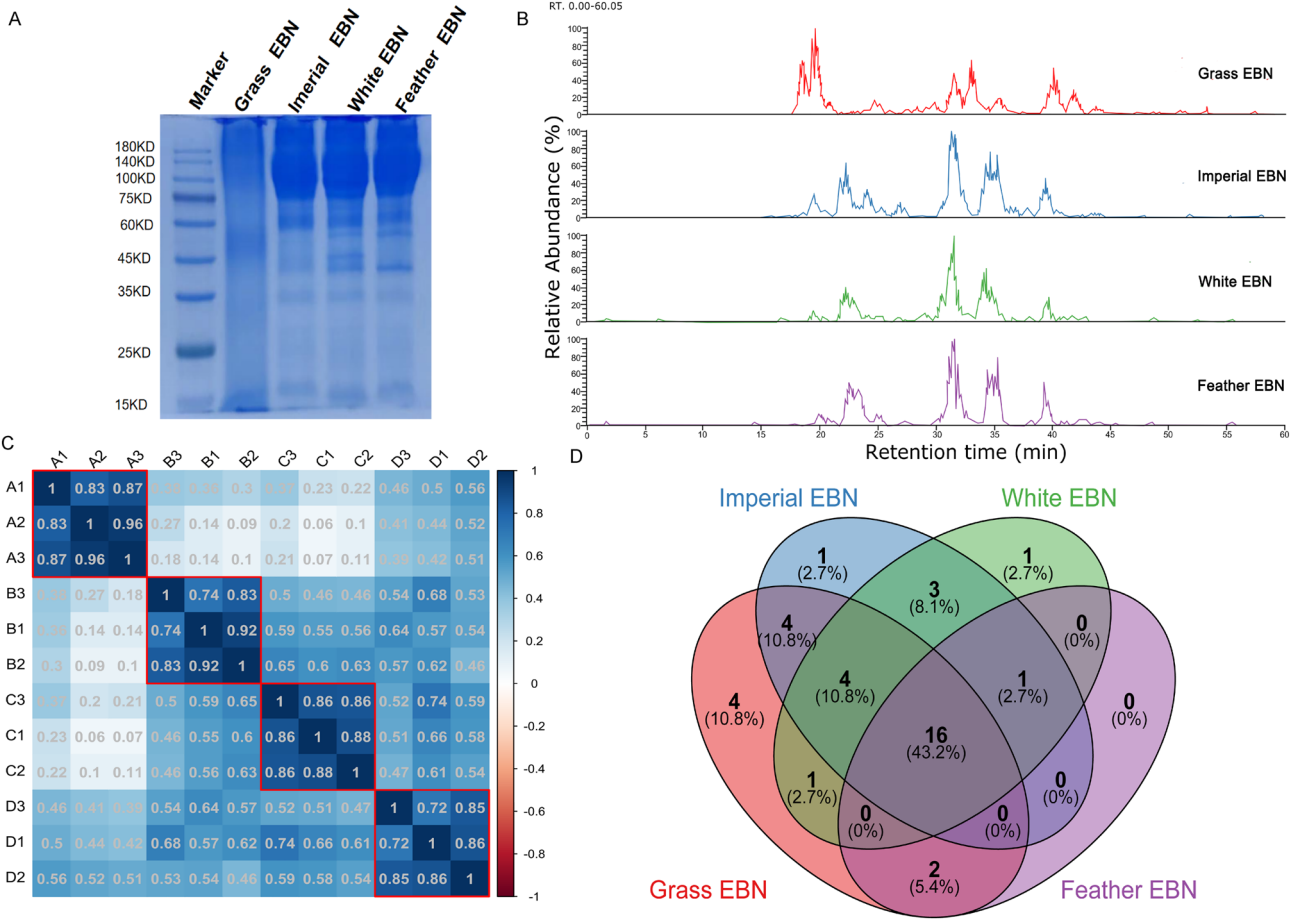
Proteomic outline of EBN's proteins. (**A**) SDS-PAGE Electropherogram of crude protein extracted from EBN samples. (**B**) Base peak chromatogram of MS^1^ (2nd test). (**C**) Pearson correlation coefficient calculated by iBAQ value. A1/A2/A3, B1/B2/B3, C1/C2/C3 and D1/D2/D3 refer to N = 3 per group.A1/A2/A3, B1/B2/B3, C1/C2/C3 and D1/D2/D3 represent Grass EBN, Imperial EBN, White EBN and Feather EBN in that order. (**D**) Venn graph shows the number of proteins identified in different EBN samples.

SDS-PAGE electrophoretogram showed that abundant proteins were extracted from EBN samples, indicating effective protein isolation (Fig. 3A). In addition, the electrophoretogram also revealed that the total protein composition of *Grass EBN* highly differs from other samples, and some protein bands exhibit between 35 and 50 kDa among all samples, supporting that there are common proteins among EBNs (Fig. 3A). After that, crude proteins were digested through filter-aided proteome preparation (FASP) and then 2 µg of digested products were used to conduct LC–MS/MS analysis (three replication for each sample). The basal peak chromatogram of MS1 (second detection) showed abundant peptide fragment sequences in the digestion products. The peptide fragment compositions of *Imperial EBN*, *White EBN*, and *Feather EBN* were relatively similar, while the peptide fragment compositions of *Grass EBN* were significantly different from the other three EBNs within the retention times of 15–25 min and 38–45 min (Fig. 3B).

### Proteomic outline of EBN's proteins

The *Chaetura pelagica* RefSeq protein database and MaxQuant were used to analyze all 12 raw MS data files by the Label-free Quantitative Proteomics Workflow (detailed in the “Materials and methods” section). A total of 421 peptides were collected and using intensity-based absolute quantitation and independent peptide segments (Supplementary Datasheet S4), 165 groups of protein were identified (Supplementary Datasheet S3). With the use of Pearson's correlation matrix, the effectiveness of experimental repetition was evaluated, and the average intra-sample repetition's Pearson correlation coefficient was greater than 0.8 (Fig. 3C), which indicated that the repeated experiments of the same sample had good reproducibility.

For further analysis, we filtered the proteins that had been successfully quantified more than twice and hit by at least one unique peptide. A total of 37 protein groups emerged, of which 31 groups were detected in *Grass EBN*, 29 groups were detected in *Imperial EBN*, 26 groups were detected in *White EBN*, and 19 groups were detected in *Feather EBN* (Supplementary table). Fascinatingly, the Venn diagram (Fig. 3D) showed that there were 16 groups of protein exhibited among all samples. Significantly, *Grass EBN* contained four characteristic proteins (V-set and immunoglobulin domain-containing protein 8, annexin A1, annexin A2, and amyloid beta A4 protein), *Imperial EBN* contained one characteristic protein (DNAJ homolog subfamily C member 3) and *Feather EBN* contained one characteristic protein (beta-hexosaminidase subunit beta), which show a potential capacity to distinguish different EBN.

### Functional analysis of proteins in EBN

**Figure 4:**
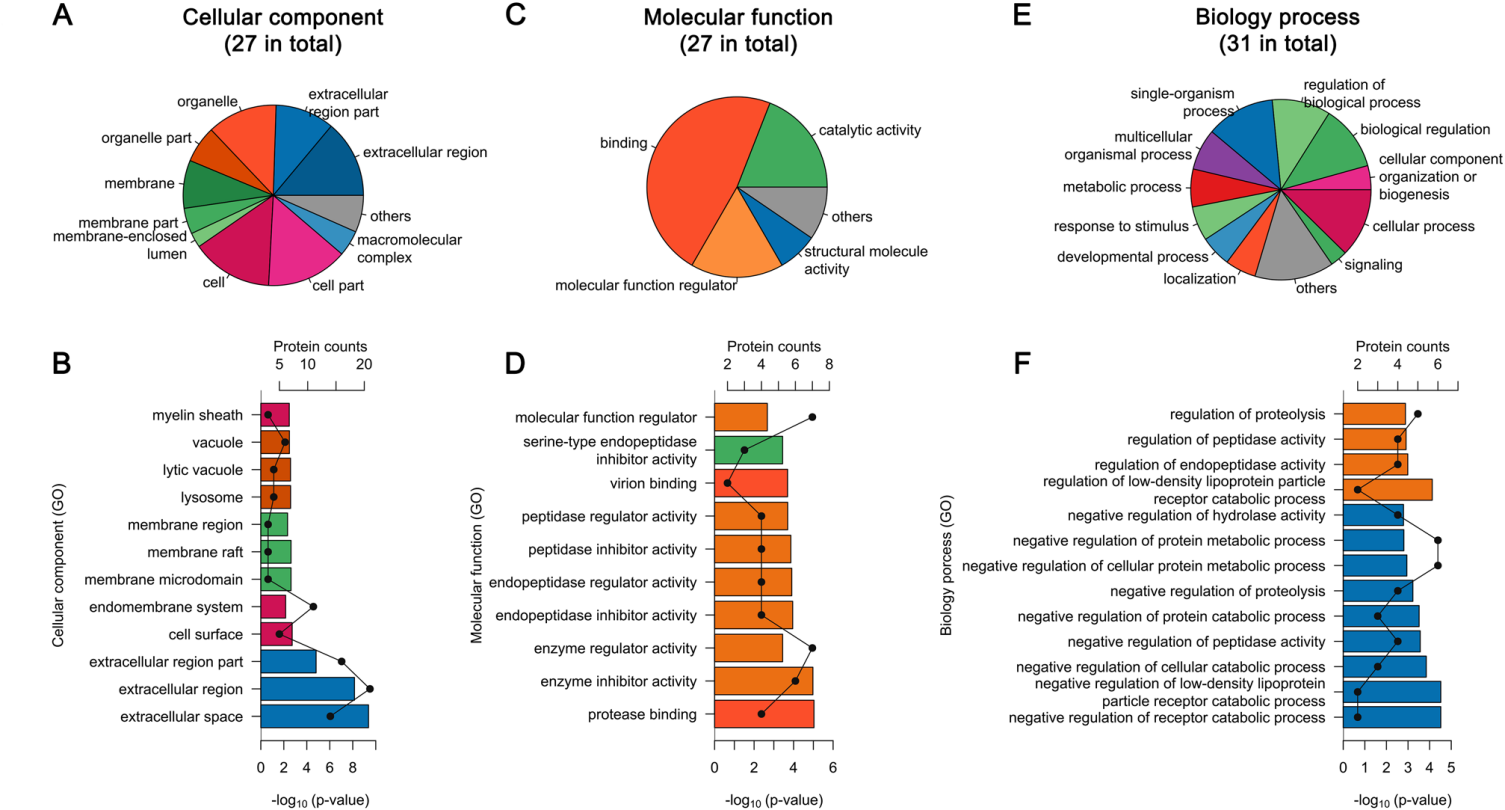
Functional analysis of EBN’s proteins. GO enrichment based on protein annotation in Gene ontology classified by cellular component (**A**,**B**), molecular function (**C**,**D**) and Biological process (**E**,**F**).

To understand the potential function of proteins in EBN, we conducted Gene Ontology (GO) analysis. GO project uses structured terms to annotate specific biological structures, so that functional annotation of gene products can be shared among species^23^. Unfortunately, there is less annotation about swiftlet proteome, thereby, the annotation of proteins identified above were referred to that of the homologous proteins of related model species *Gallus gallus*. The three categories of GO annotation are molecular function, cellular components, and biological processes. The analysis revealed that 27 of 37 proteins had annotation for cellular components, 27 proteins had annotation for molecular functions, and 31 proteins had annotation for biological processes. (Fig. 4A,C,E). Secondary annotation in different categories showed that proteins of EBN were distributed in extracellular, organelle, cell membrane, and cell–matrix (Fig. 4A), the main molecular functions were binding, catalytic activity, molecular function regulator and structural molecular activity (Fig. 4C), and the proteins are involved in a variety of biological processes (Fig. 4E). GO enrichment also revealed that proteins of EBN were significantly distributed in the extracellular, cell surface, cell membrane, and lysosome, which suggests that proteins of EBN belong to secretory proteins (Fig. 4B). In addition, proteins of EBN significantly showed a regulatory effect of various proteases in molecular function (Fig. 4D), and negative regulation characteristics in the biological process of protease regulation (Fig. 4F). However, whether these functions are responsible for the therapeutic effects remains to be further studied.

### Quantitative analysis of proteins in EBN

**Figure 5:**
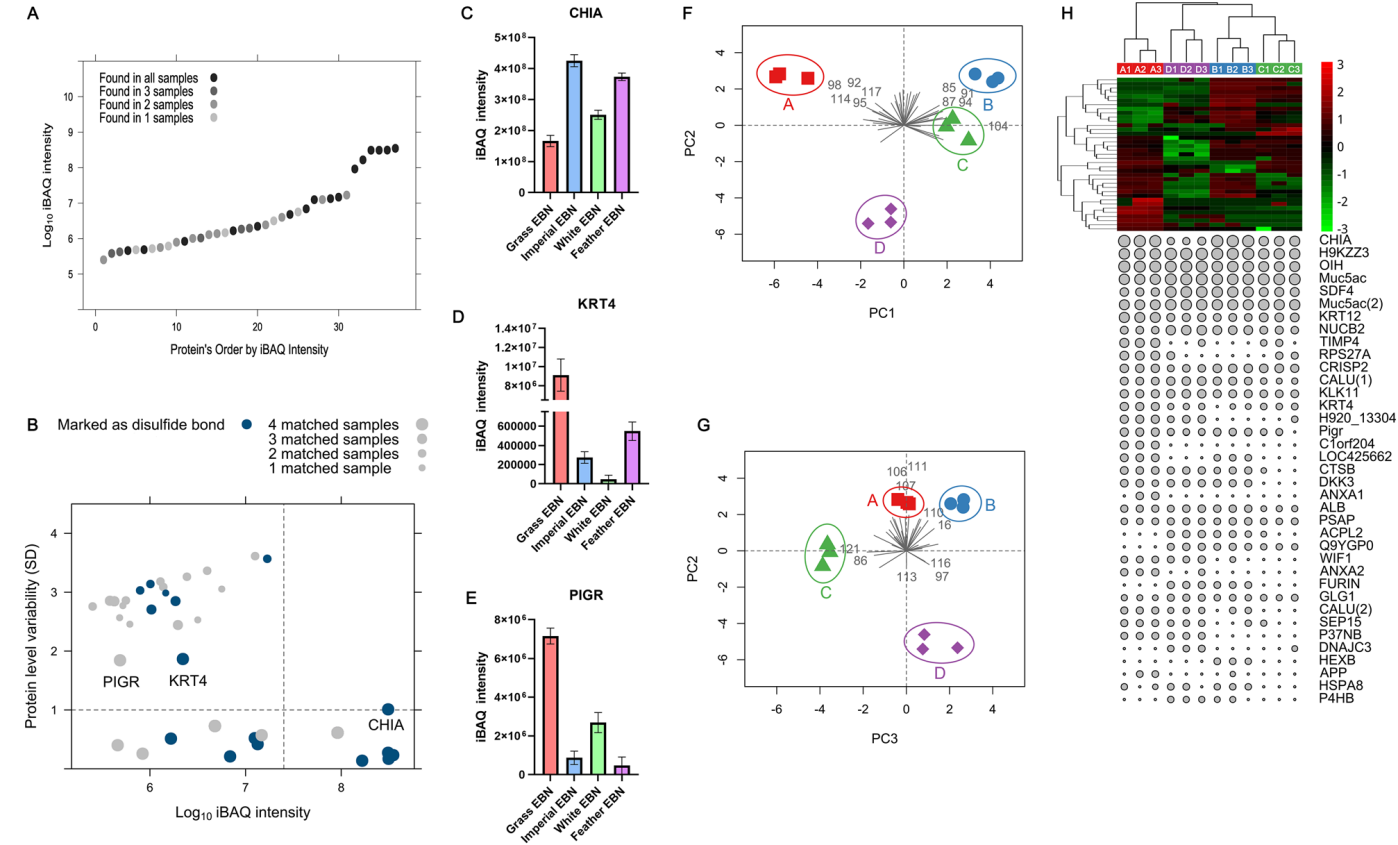
Quantitative analysis of EBN’s proteins. (**A**) Scatter diagram of Protein concentration ranged by iBAQ intensity from low to high. Protein was detected in all samples marked with black, and protein was detected in some samples marked with gray. (**B**) Scatter diagram of protein concentration and the difference between samples. Proteins with disulfide bonds were highlighted in blue. (**C**–**E**) Histogram of the iBAQ intensity of CHIA, KRT4, and PIGR in EBN samples. (**F**,**G**) Principal component analysis based on protein composition and quantitation. A, B, C, and D refer to *Grass EBN*, *Imperial EBN*, *White EBN*, and *Feather EBN* respectively. (**H**) Hierarchical clustering based on label-free proteome quantification. The size of the dots below refers to the iBAQ intensity of each detection. A1/A2/A3, B1/B2/B3, C1/C2/C3 and D1/D2/D3 refer to N = 3 per group.A1/A2/A3, B1/B2/B3, C1/C2/C3 and D1/D2/D3 represent Grass EBN, Imperial EBN, White EBN and Feather EBN in that order.

Next, we performed a quantitative analysis to compare the protein composition of EBN samples. iBAQ value calculated by Maxquant software (detailed in “Materials and methods”) is selected as the quantitative index, which can accurately quantify the relative abundance of all proteins within the same sample and calculation process^24^. The analysis revealed that the iBAQ values of different proteins varied greatly, and the dynamic range reached three orders of magnitude, indicating the complexity of protein composition in EBN (Fig. 5A,B). Significantly, there are three proteins (CHIA, KRT4, and PIGR) detected in all samples and showing greatly diverse intensity within different samples, which exhibit potential value in differentiating EBNs (Fig. 5B–E). Furthermore, there are 6 proteins detected in all samples and exhibiting significantly higher density than others (Supplementary Datasheet S2), including two mucin-5AC-like proteins (MUC5B), lysyl oxidase homolog 3 protein (LOXL2), acidic mammalian chitinase-like protein (CHIA), ovoinhibitor-like protein (OIH) and 45 kDa calcium-binding protein (CAB45) (Table 1 and Supplementary Datasheet S1). Annotation revealed that except CAB45 which localizes to the secretory pathway of mammalian cells^25^, all proteins are marked as secretory proteins and rich in disulfide bonds which contribute to the folding and stability of extra-cytoplasmic proteins^26^. In addition, the five common proteins with the highest iBAQ intensity (two MUC5B, LOXL2, CHIA and CAB45) belong to glycoprotein, in which MUC5B contains more than ten N-glycosylation sites and is reported to protect the mucous membrane^27^.

**Table 1:** Information of six common proteins significantly exhibiting high iBAQ intensity.

Protein description	Accession number	Uniprot ID	Symbol	Marked as secretory protein	With disulfide-bond keyword	Marked as glycoprotein
Lysyl oxidase homolog 3	XP_010006484	E1C3U7	LOXL2	+	+	+
Mucin-5AC-like, partial	XP_009994736	Q98UI9	MUC5B	+	+	+
Acidic mammalian chitinase-like, partial	XP_010006620	F1NMM2	CHIA	+	+	+
Ovoinhibitor-like	XP_010000294	P10184	OIH	+	+	−
Mucin-5AC-like, partial	XP_010002210	Q98UI9	MUC5B	+	+	+
45 kDa calcium-binding protein	XP_010003465	Q5ZKE5	CAB45	−	−	+

To compare the protein composition of different EBNs, we established a 12 * 37 matrix according to protein iBAQ values and its source. The principal component analysis shows that the first 3 components can explain more than 80% of the total variance (Fig. 5F,G). From the PC1 vs PC2 graph, we can see that repeats of each sample are clustered together and the *White nest* and *Imperial nest* are clustered closer in the first two components. We can also find that *Grass EBN* is separated apart in the PC1 direction, *Feather EBN* has separated apart in the PC2 direction while *White EBN* and *Imperial EBN* are separated apart in the PC3 direction in the PC2 vs PC3 direction (Fig. 5F,G).

The heat map creates an intuitive interface of differential proteins in four samples (Fig. 5H). We can infer from the graph below that *Grass EBN*, *White EBN* and *Imperial EBN* are rich in protein, while the poverty of protein content of *Feather nest* reveals that *Aerodramus Maximus* need to construct their nest by feather and saliva. Hierarchical clustering showed that four EBN samples were divided into three clusters, in which *Imperial EBN* and *White EBN* exhibited similar protein composition, clustering to the same branch. *Grass EBN* and *Feather EBN* were distributed into the other two separated branches, while the protein composition of *Feather EBN* was relatively similar to that of *Imperial EBN* and *White EBN*, which is consistent with the genetic relationship in the phylogenetic tree (Fig. 2C).

## Discussion

EBN is a well-known biological product made from the saliva of swiftlets and has been used in China for hundreds of years, mainly from the Philippines in the east to the caves of deserted islands off the coast of Myanmar in the west, mostly in Indonesia, Malaysia, Sandakan, Singapore and Thailand in Southeast Asia and the islands in the South China Sea^28^. EBN is rich in sugars, organic acids, free amino acids and sialic acid, which is a characteristic substance that nourishes the Yin, moistens dryness and tonifies the middle and vital energy^29–31^. As a high-grade tonic, it is very popular among domestic and foreign consumers^32^. Due to the restriction order on the collection of wild EBN and the ineffective breeding and processing of EBN, the low yield has led to the high price of EBN, which has induced the production of counterfeit EBN and caused confusion in the EBN trade^33^. Currently, the quality evaluation of EBN in the trade relies mainly on morphological identification, which is non-quantitative and subjective. Previous studies have found that the protein content of EBN is more than 50% of its composition^34^.

The current state-of-the-art identification methods for EBN authentication are based on sophisticated techniques of genetics, immunochemistry, spectroscopy, chromatography and gel- electrophoresis to detect various types of adulterants in EBN^35^. For example, in a study by Lee and Seow^36^ et al., the former distinguished EBN from West Malaysia (WM) and East Malaysia (EM) based on amino acid profiles by high performance liquid chromatography (HPLC) combined with a multivariate approach, while the latter determined the amino acid composition of EBN samples by gas chromatography-mass spectrometry (GC–MS) for stoichiometric analysis. However, fewer studies have focused on the protein composition of EBN from different origins, and future proteomics-based identification methods will be more reliable and need to be urgently developed. In this study, we applied the label-free proteomic technique to study the different EBN samples collected from three major producing countries, and our results reveal that a total of 37 groups of protein are identified from EBN samples, which helps to understand the protein composition of EBN. In addition, some characteristic proteins were detected in specific EBN samples, which shows a potential capacity to distinguish different EBNs. For example, *Grass EBN* contained four characteristic proteins (V-set and immunoglobulin domain-containing protein 8, annexin A1, annexin A2 and amyloid beta A4 protein), *Imperial EBN* contained one characteristic protein (DNAJ homolog subfamily C member 3) and *Feather EBN* contained one characteristic protein (beta-hexosaminidase subunit beta) (Fig. 3D, Supplementary table).

The material basis of the therapeutic efficacy of EBN is still unknown, and there are many incomplete elucidations in the view of elemental properties^37,38^, amino acid composition^39^ and sialic acid content^40,41^. Here, we conduct GO analysis using 37 identified proteins and attempt to understand the function of EBN proteins. Our results reveal that the proteins of EBN mainly belong to secretory proteins, which is in accord with the origin of EBN (Fig. 4A,B). Besides, the proteins of EBN show a wide variety of enzyme activities (Fig. 4C,D) and play a role in various negative-regulation in biological process (Fig. 4E,F). But note, whether the enzyme activities of proteins are retained in dried EBN or EBN products need to be further studied.

There are many categories of EBN according to colors, producing countries, and processing and previous studies have reported the chemical composition of EBN in different colors, production sites, geographical origin, and even biological origin, including elemental properties, nitrite and nitrate contents, sialic acid content, amino acid profiling, and total phenolic content^39,42–45^. In this study, we conduct a quantitative analysis of 37 identified proteins based on intensity-based absolute quantification algorithm^24^ applied in the protein identification process. Significantly, there are six common proteins (two MUC5B, LOXL2, CHIA, OIH and CAB45) remarkably exhibiting high iBAQ intensity, indicating high content of these proteins in EBN, which could be quantitative indexes in quality control (Fig. 5A). The contents of the three proteins (CHIA, KRT4 and PIGR) varies greatly among different samples showing a capacity to distinguish different EBNs (Fig. 5B–E). However, we are unable to verify the exhibition of these proteins with other techniques, such as western blot and ELISA because of lacking specific antibody. Therefore, many efforts are in urgent need of developing the specific antibody against EBN proteins.

EBN made from the saliva of swiftlet could be regarded as biological secretions, just like milk. Proteomic techniques have been applied to decode the protein composition of milk from different species, evaluating the difference in nutritive value^46–48^. As for EBN, there is no study about the relatedness of the biological origin and protein composition of EBN. Here, we creatively combined genetic and proteomic technique to reveal the association between biological origin and protein composition of EBN. The biological origin of *Grass EBN/Imperial EBN/White EBN* and *Feather EBN* are identified as *Apus pacificus*/*Aerodramus fuciphagus germani*/*Aerodramus fuciphagus* and *Aerodramus maximus* respectively (Fig. 2A–C). In addition, hierarchical clustering based on protein composition and quantification reveals that *Imperial EBN* and *White EBN* exhibit similar protein composition, and *Feather EBN* is relatively similar to that of *Imperial EBN* and *White EBN* (Fig. 5H), which is consistent with the genetic relationship in the phylogenetic tree (Fig. 2C). The coincident relatedness of different EBN in the view of genetic origin and protein composition suggests that the biological origin of EBN should be an important factor in quality control.

## Conclusion

The composition consists of more than 50% protein, but limited studies are focusing on the protein composition of EBN. Here, we collected four EBNs from three major producing countries and applied Label-free proteomic technique to decode the protein composition of EBN. A total of 37 proteins were identified from EBN samples, and six proteins detected in all samples with high content and characteristic proteins detected in specific EBN show potential capacity in EBN quality control and distinction. GO analysis reminds us that the proteins in EBN exhibit varieties of enzyme activities, but whether it retains in dried EBN or EBN products need further study. Quantitative studies also reveal that *Imperial EBN* and *White EBN* exhibit similar protein composition, and *Feather EBN* is relatively similar to that of *Imperial EBN* and *White EBN*, which is consistent with the relatedness of the EBN biological origin. Collectively, our study decodes the protein composition of EBNs from different origins, which helps to formulate a quantitative and reliable quality evaluation system, and the identified proteins shed new light on efficacy study.

## Materials and methods

### Sample collection and sample processing

EBN samples were provided from TUNGHONG MEDICINE Co., Ltd, which collected samples from China, Vietnam and Indonesia respectively (Fig. 1). To decode the proteomic components of EBN, crude proteins were extracted from sample powders through soaking in 0.2 M urea for 4 h, ultrasonic processing for 1 h, and then and centrifuging (10,000*g*) for 15 min.

### SDS-PAGE electrophoretogram

After freeze-thawing, 200 μL SDT (4% SDS, 100 mM DTT, 150 mmtris-hcl pH 8.0) were added to the extracted bird's nest protein solutions, followed by boiling water bath for 5 min, ultrasonic cracking, centrifugation for 45 min, 10 μL samples were taken for electrophoresis. After electrophoresis, the gel was stained with 0.25% Coomassie bright blue and destained with decoloring Solution (5% ethanol and 10% acetic acid).

### Genetic identification

According to our previously developed methods^22^, total genomic DNA was extracted from EBN samples, and nest PCR technique was performed to amplify cytb gene fragments with the following primers. Forward primer: 5′-TAGCTAGGATCTTTCGCCCT-3′. Reverse primer: 5′-GGCATATGCGAATARGAARTATCA-3′. Amplified fragments were sequenced, and then the sequencing results were imported to BLAST to hit the most similar sequences. MAFFT v7.308^49^ and Aliview software^50^ were used to align and adjust the sequences in the dataset. The sequence information of the sample was recorded in GenBank. The accession numbers of *Grass EBN* is MW273377, *Imperial EBN* is MW273380, *White EBN* is MW273379, and *Feather EBN* is MW273378. Genetic distance was calculated using Kimura-2-parameter model in MEGA v7^51^. For phylogenetic analysis. A Neighbor-Joining tree was constructed using a dataset, including amplified sequences and sequences of cytb genes belonging to *Apodidae*. The Kimura 2-parameter model was selected to build the neighbor-joining tree and sequences of cytb gene of *Amazilia tzacatl* was selected as outgroup. The reliability of the topology was evaluated with bootstrap values obtained 1000 times. FigTree v1.4.2 was used to visualize the phylogenetic tree.

### Extraction of EBN crude protein for LC–MS/MS analysis

Using 0.2 M urea, 4 h of soaking, 1 h of ultrasonic processing, and 15 min of centrifuging at 10,000 rpm were used to extract crude proteins from sample powders. Dithiothreitol (DTT) was added to 100 g of the samples for LC–MS/MS analysis at a final concentration of 100 mM before the samples were chilled and reduced to 95 °C for 5 min. After adding 200 μL of UA buffer (8 M urea, 150 mM Tris–HCl, pH 8.0) to the samples, an ultrafiltration filter (30 kDa cutoff) was placed onto the samples. The mixture was then centrifuged at 14,000*g* for 30 min before being washed once more with 200 μL of UA buffer. The filter was then added to 100 μL of iodoacetamide solution (50 mM iodoacetamide in UA buffer) for alkylate, mixed on a shaker for 1 min at 600 rpm after 30 min of incubation in the dark at room temperature, and then centrifuged once more for 20 min at 14,000*g*. Following two cycles of centrifugation at 14,000*g* for 30 min, the filter was added to 100 μL of UA buffer. After that, the filter was added to three volumes of 25 μL of 25 mM ammonium bicarbonate, and each addition was centrifuged at 14,000*g* for 30 min. After that, 40 μL of trypsin buffer (3 g trypsin in 40 μL 25 mM ammonium bicarbonate) was added, the filter was shaken at 600 rpm for 1 min, and it was then incubated at 37 °C for 16–18 h. After that, swap out the filter's used collecting tube for a new one before centrifuging it for 20 min at 14,000*g*. The filter was added to 25 μL of 25 mM ammonium bicarbonate, agitated for 1 min at 600 rpm, and then centrifuged as usual at 14,000*g*. The filtrate was subsequently collected, desalted on a C18 Cartridge column (EmporeTM SPE Cartridges C18 (standard density), bed I.D. 7 mm, volume 3 mL, Sigma), lyophilized to dryness, and reconstituted in 60 μL of 0.1% (v/v) trifluoroacetic acid. At OD 280, the peptide's ultimate concentration was measured.

### LC–MS/MS analysis

MS experiments were performed using a Q-Exactive (Thermo Fisher Finnigan) mass spectrometer tandem to EASY-nLC (Proxeon Biosystems, now Thermo Fisher Scientific) via a nanoelectrospray source. 2 μg peptide solved in buffer A (2% acetonitrile and 0.1% Formic acid) was automatically loaded on a trap column (Thermo Scientific Easy Column, SC001 traps 150 μm*20 mm) and was separated on a C18-reverse phase column (Thermo Scientific Easy Column, SC200 150 μm*100 mm) over a 120 min linear gradient of buffer A and B from 0 to 100% (100 min to 45%, 8 min to 100% and kept 100% for 12 min) in buffer B (84% acetonitrile, 0.1% formic acid) at a flow rate of 400nL/min controlled by IntelliFlow technology. The survey scan (300–1800 m/z) for HCD fragmentation was used to select the top 20 most abundant precursor ions dynamically for MS data acquisition. Predictive Automatic Gain Control is used to determine the target value (AGC). The dynamic exclusion lasted for 25 s. HCD spectra were obtained with a resolution of 17,500 at m/z 200, while survey scans had a resolution of 70,000 at this wavelength. The underfill ratio, which indicates the smallest percentage of the target value expected to be obtained at maximum fill time, was set at 0.1% and the normalized collision energy was 30 eV. Peptide recognition mode was activated while the instrument was being used. For each sample, MS studies were carried out three times.

### Protein identification

All 12 raw MS data files were analyzed using MaxQuant version 1.5.2.8^52^ and peptide identification was carried out with the Andromeda search engine. Briefly, fragmentation spectra was searching against a combined database that were the *Chaetura pelagica* RefSeq protein database (from NCBI, forward and reverse sequences), and the common contaminants database from MaxQuant. The tolerance for fragment mass was initially set at 20 ppm, while the maximum tolerance for precursor mass was set at 7 ppm for the initial search and 20 ppm for the main search. During the search, a minimum of 7 amino acids and a maximum of 2 missed cuts were permitted. Protein N-terminal acetylation and methionine oxidation database searches were set as the variable modifications, with carbamidomethylation of cysteines as the fixed modification. Using reverse sequences as a decoy database, the cutoff of false discovery rate (FDR) for proteins and peptides identification was set to 0.01. To estimate protein abundance, the discovered peptides were subjected to the intensity-based absolute quantification technique of MaxQuant.

### Statistical analysis

ProteinGroups.txt, the MaxQuant output file, was imported using an internal script into a SQLite database. Protein groups detected in contaminants' or reverse databases were eliminated, but protein groups supported by positive scores, positive overall iBAQ intensities, and at least one unique peptide were included. All proteins were identified as being either swiftlet- or insect-sourced in their original database. Then, proteins that made up the first majority of each protein group were chosen as identified proteins. R software was used for the statistical study, primarily the stats (version 3.2.2), complot (version 0.77), heatmap (version 1.0.8), or lattice packages (version 0.20-33). Inkscape was used to create the Venn diagram and any necessary graphical embellishments. The proteins were specifically measured using the log iBAQ intensities. Pearson's correlation matrix was used to assess the consistency of the experimental replication. Each sample's number of identifications and the overlapped or identical proteins were compared with a Venn diagram to determine the number of identified proteins that had been successfully detected in more than one repetition of each sample. With the help of the xyplot function, level ranges and diversity of median log iBAQ intensities were depicted. While PCA converts multivariate data into non-correlated variables, namely principle components and splits each observation according to the least components, hierarchical clustering joins comparable observations based on how far off they are from one another. Multivariate analysis, Euclidean distance, and Euclidean space were performed on scaled data.

### Functional analysis

As annotations from the RefSeq database limiting, identified proteins were re-annotated with Uniprot database and Gene Ontology (GO) database. Detailed gene names or other annotations were acquired from homologous reference proteins and total proteins' functional outline was acquired from GO annotation. Briefly, protein sequences were done BLAST against *Gallus gallus*'s reference proteome (proteome id: UP000000539_9031, with swiss-prot’s annotation) and the best match with e-value under 10^–5^ was chosen as the homologous reference proteins. GO annotation was mainly done using BinGO plug-in (version 3.0.3). Ontology file (obo file) and annotation file (gaf file, species *Gallus gallus*) was downloaded from Gene Ontology Consortium official site. Reference proteins' Uniprot ID were used to acquire GO annotation and the whole annotation of *Gallus gallus* was set as reference background. GO enrichment analysis for protein function classes was performed using a hypergeometric test with Benjamini's false discovery rate (FDR) correction.

### Supplementary Information

Supplementary Information 1.

## Data Availability

The mass spectrometry proteomics data have been deposited to the ProteomeXchange Consortium (http://proteomecentral.proteomexchange.org) via the iProX partner repository^53, 54^ with the dataset identifier PXD042717.
